# On nature-inspired design optimization of antenna structures using variable-resolution EM models

**DOI:** 10.1038/s41598-023-35470-4

**Published:** 2023-05-24

**Authors:** Slawomir Koziel, Anna Pietrenko-Dabrowska

**Affiliations:** 1 Engineering Optimization and Modeling Center, Reykjavik University, 102 Reykjavik, Iceland; 2 Faculty of Electronics, Telecommunications and Informatics, Gdansk University of Technology, 80-233, Gdansk, Poland

**Keywords:** Computational science, Electrical and electronic engineering

## Abstract

Numerical optimization has been ubiquitous in antenna design for over a decade or so. It is indispensable in handling of multiple geometry/material parameters, performance goals, and constraints. It is also challenging as it incurs significant CPU expenses, especially when the underlying computational model involves full-wave electromagnetic (EM) analysis. In most practical cases, the latter is imperative to ensure evaluation reliability. The numerical challenges are even more pronounced when global search is required, which is most often carried out using nature-inspired algorithms. Population-based procedures are known for their ability to escape from local optima, yet their computational efficiency is poor, which makes them impractical when applied directly to EM models. A common workaround is the utilization of surrogate modeling techniques, typically in the form of iterative prediction-correction schemes, where the accumulated EM simulation data is used to identify the promising regions of the parameter space and to refine the surrogate model predictive power at the same time. Notwithstanding, implementation of surrogate-assisted procedures is often intricate, whereas their efficacy may be hampered by the dimensionality issues and considerable nonlinearity of antenna characteristics. This work investigates the benefits of incorporating variable-resolution EM simulation models into nature-inspired algorithms for optimization of antenna structures, where the model resolution pertains to the level of discretization density of an antenna structure in the full-wave simulation model. The considered framework utilizes EM simulation models which share the same physical background and are selected from a continuous spectrum of allowable resolutions. The early stages of the search process are carried out with the use of the lowest fidelity model, which is subsequently automatically increased to finally reach the high-fidelity antenna representation (i.e., considered as sufficiently accurate for design purposes). Numerical validation is executed using several antenna structures of distinct types of characteristics, and a particle swarm optimizer as the optimization engine. The results demonstrate that appropriate resolution adjustment profiles permit considerable computational savings (reaching up to eighty percent in comparison to high-fidelity-based optimization) without noticeable degradation of the search process reliability. The most appealing features of the presented approach—apart from its computational efficiency—are straightforward implementation and versatility.

## Introduction

The development of modern antenna systems is a complex endeavor facing numerous challenges. The majority of these result from increasing performance requirements associated with the newly developed areas such as internet of things (IoT)^1^, microwave imaging^2^, body area networks^3^, 5G wireless communications^4,5^, or remote sensing^6^, as well as additional functionalities required by specific applications (broadband^7^ and multi-band operation^8^, MIMO operation^9^, reconfigurability^10^, polarization diversity^11^, beam scanning^12^, enhanced gain^13^). An additional difficulty arises due to miniaturization demands: compact antennas are essential for mobile communications^14^, IoT^15^, as well as wearable and implantable devices^16,17^. At the same time, downsizing generally exerts adverse effect on electrical and field performance of the radiators^18^, and trade-off designs have to be devised that ensure required functionality while satisfying geometrical constraints. Meeting the aforementioned performance demands fosters the development of unconventional and often topologically complex antenna structures that feature a variety of additional components, e.g., stubs^19^, slots^20^, shorting pins^21^, defected ground structures^22^, or multi-layer implementations^23^. The recent introduction of metamaterials (e.g., in the form of metasurfaces) enables the design of more sophisticated antenna geometries featuring improved performance with respect to gain, radiation properties, reduced size, or improved element isolation for multi-radiator systems^101–104^. On the other hand, reliable evaluation of geometrically involved antenna structures can only be realized using full-wave electromagnetic (EM) analysis. Consequently, EM simulation has become ubiquitous as a design tool, which is indispensable at all stages of the design process, including topology evolution, parametric studies, as well as design closure (i.e., final tuning of geometry parameters).

Ensuring the best possible performance of contemporary antennas requires meticulous EM-driven adjustment of their parameters. Given the topological complexity, conventional enhancement methods involving equivalent network models or experience-driven parametric studies can only yield sub-optimal designs, and are generally unsuitable for handling multiple design goals, conditions on electrical performance figures, and multiple parameters. Instead, rigorous numerical optimization is recommended^24,25^. Probably the most serious bottleneck thereof constitutes the inflated computational cost that is problematic even for local parameter adjustment (e.g., gradient-based^26^). On the other hand, global search is often necessary in many practical cases. These include tasks that exhibit multimodality (the existence of several local optima), such as optimization of metamaterials (e.g., frequency-selective surfaces^27^), array pattern synthesis for minimum sidelobe levels^28,29^, as well as all sorts of problems where decent initial designs may not be available. The last category includes the structures incorporating various geometrical alterations, introduced to implement additional functionalities (e.g., band notches^30^, multiple operating bands^31^) or permit size reduction^32^, but also structures re-designed with respect to center frequencies and material parameters being distant from those at the available design. Needless to say, in computational terms, global optimization is considerably more expensive than local procedures.

Without a doubt, the most popular techniques for global optimization are nature-inspired algorithms^29,33,34,105,106^. Their origin dates back to nineteen-eighties (e.g., genetic algorithms^35^, genetic programming^36^, ant systems^37^, evolutionary algorithms^38^), yet some early population-based methods for continuous optimization, specifically evolutionary strategies, were conceived in 1960s^39^. A significant progress has been observed in 1990s with the development of techniques such as particle swarm optimizers (PSO)^40^, or differential evolution (DE)^41^. Since early 2000s, nature-inspired methods have been dominating global optimization. Recently, a number of new methods of this class has been growing rapidly (e.g., firefly algorithm^42^, harmony search^43^, grey wolf optimization^44^, as well as many other algorithms^45–51^), yet the practical differences between them seem to be minor. Nature-inspired algorithms act upon the sets of candidate solutions (referred to as a population^52^, swarm^53^, pack^54^, etc.), the members of which (individuals, agents, particles, etc.) exchange information and produce new data using exploitative and exploratory operators^55^. This allows for locating the encouraging regions of the design space and increase the likelihood of escaping from local optima. Consequently, the algorithms of this class exhibit global search capability^56,57^. The implementation of nature-inspired methods is straightforward, however their cost effectiveness is far from satisfactory: depending on the difficulty of the problem at hand and the number of system parameters, a single search may involve anything between a few hundred and several thousands of merit function evaluations. Needless to say, this level of costs is prohibitive when the responses of the antenna under study are simulated using full-wave analysis.

Given the inferior CPU efficiency of population-based methods, their practical utility for antenna optimization is quite limited. Possible scenarios include low evaluation cost of the objective function (e.g., pattern synthesis with the use of analytical array factor models^58,59^), relatively low cost of EM analysis (simple structures with the simulation times of seconds), or parallel implementations. The latter depends upon the availability of sufficient computational resources and software licensing. In other cases, i.e., expensive EM simulations are utilized for antenna evaluation, a workaround the high cost issue is the employment of surrogate modeling methods^60–62^, mainly data-driven (e.g., kriging^63^, Gaussian Process Regression, GPR^64^, artificial neural networks^65^). The surrogate model allows for accelerating the search process by replacing costly EM simulations. In practice, the construction of the metamodel is often an iterative process, where surrogate-assisted predictions are followed by model refinement, using the accumulated high-fidelity data. The infill samples are allocated to enable parameter space exploration (when the improvement of global accuracy of the surrogate is required) or exploitation (when the primary purpose is optimum identification)^66^. Other possible approaches include machine learning methods^67^, often involving sequential sampling techniques^68^. Surrogate-assisted pre-screening of the parameter space is also occasionally employed^69^. Despite their potential merits, the use of surrogate modeling methods for global optimization of antenna structures is impeded by the curse of dimensionality but also significant nonlinearity of antenna frequency characteristics. In practice, utilizing general-purpose modeling techniques poses problems for devices featuring more than a few geometry parameters^70–72^. The mitigation methods include domain confinement^73,74^, incorporation of variable-resolution EM simulations^75^, as well as the response feature methodology^76^. The latter benefits from a weakly-nonlinear dependence of the coordinates of appositely singled out characteristic points of antenna responses on the geometry parameters (as opposed to the complete responses), which allows—upon reformulation of the design problem with the use of response features—for a faster convergence of the optimization process^77^, or a reduction of the number of training samples (in the context of surrogate modeling^78^).

The techniques outlined in the previous paragraph address certain issues of global EM-driven optimization of antenna systems, yet they suffer from the number of problems on their own, lack versatility, and are relatively complex to implement. Perhaps the simplest speedup approach would be the incorporation of variable-fidelity models. In the realm of local design of high-frequency components, this has been mostly done at two levels of fidelity (e.g., equivalent circuit models versus EM simulations, e.g.^79,80^) or resolution (e.g., coarse- and fine-discretization EM analysis^81,82^). Utilization of model resolutions from a continuous spectrum constitutes a more attractive option. It has been applied for expediting local antenna design optimization in^107,108^, also in combination with various reliability enhancement mechanisms^109,110^. Whereas in the context of nature-inspired algorithms, various fidelity adaptation schemes were investigated in^83^; however, using mostly analytical objective functions. In this work, we investigate potential benefits of employing variable-resolution EM models for global optimization of antennas using nature-inspired algorithms. In pursuit of implementation simplicity but also generality, an automated procedure utilizing EM simulation models chosen from a continuous spectrum of resolutions is developed. In our work, model resolution is controlled by the discretization density of the antenna structure at hand. Starting from the lowest admissible resolution, determined as rendering all relevant features of the system characteristics (e.g., the resonances), the model fidelity is gradually increased to reach the high-fidelity level at the conclusion of the algorithm run. Two research questions arise: (i) how much faster the global design optimization of antenna structures may be carried out using variable-resolution EM models than the procedure executed in a single-fidelity regime, and (ii) to what degree the design quality is going to deteriorate with respect to the algorithm employing solely high-resolution EM simulations. The speedup versus quality trade-offs are investigated for various resolution adjustment profiles, and using particle swarm optimizer (PSO) (being a commonplace population-based algorithm). The benchmark set includes four microstrip antennas of distinct characteristics. The obtained results demonstrate that appropriate resolution adjustment enables considerable savings (up to nearly eighty percent as compared to high-fidelity-based optimization) without compromising reliability of the search process. The attractive features of the presented approach, as compared to alternatives discussed earlier in this section, include computational efficiency but also easy implementation and versatility.

The novelty and technical contributions of the paper include: (i) a conceptual development of an algorithmic framework which incorporates variable-resolution EM simulations into nature-inspired antenna optimization, (ii) development of a resolution management scheme utilized by the proposed global search procedure based on the algorithm convergence status, (iii) demonstrating of significant CPU savings (up to 70%) over the single-resolution approach obtained without noticeable degradation of the solution quality. To the best knowledge of the authors, it is the first time that the multi-resolution simulation models have been used in conjunction with nature inspired algorithms for high-frequency design.

## EM-driven design of antennas variable-resolution models

In this section, we recall the formulation of EM-driven antenna optimization task and discuss variable-resolution models. In particular, we explain the process of establishing a suitable range of EM simulation model fidelities that can be used in antenna optimization. It is illustrated using specific examples of microstrip antennas.

### EM-driven design of antenna structures

Rigorous numerical optimization has become ubiquitous in antenna design, although traditional parameter tuning methods, mainly parameter sweeping guided by engineering experience, are still widely used by the designers. Formal optimization requires a definition of a performance metric, which is normally a scalar function of adjustable parameters (typically, antenna dimensions), but might also be vector-valued in the case of multi-objective design. In this work, we do not consider multi-objective design^84^, therefore, at the presence of multiple objectives, they are assumed to be aggregated in some form (weighted sum approach^85^) or cast into design constraints with the user-defined acceptance thresholds (cf. examples below).

Let ***x*** denote a vector of adjustable parameters of the antenna of interest, which are normally its geometry parameters. The parameter adjustment task is defined as

1 $${\boldsymbol{x}}^{*} = \arg \mathop {\min }\limits_{{\boldsymbol{x}}} U({\boldsymbol{x}})$$

where x^*^ denotes the optimal vector to be found, and *U* stands for the scalar merit function that quantifies the designer’s view concerning the design quality. In particular, it should be defined so that better designs correspond to lower values of *U*(***x***). In general, the process (1) is subject to inequality constraints *g*_*k*_(***x***) ≤ 0, *k* = 1, …, *n*_*g*_, and equality constraints *h*_*k*_(***x***) = 0, *k* = 1, …, *n*_*h*_. As the antenna structures are typically evaluated using full-wave electromagnetic (EM) simulations, explicit handling of constraints is usually impractical. An alternative is a penalty function approach^86^, where the optimization task (1) is replaced by

2 $${\boldsymbol{x}}^{*} = \arg \mathop {\min }\limits_{{\boldsymbol{x}}} U_{P} ({\boldsymbol{x}})$$

In (2), the function *U*_*P*_ constitutes a linear combination of the penalty terms and the original objective function *U*. We have

3 $$U_{P} ({\boldsymbol{x}}) = U({\boldsymbol{x}}) + \sum\nolimits_{k = 1}^{{n_{g} + n_{h} }} {\beta_{k} c_{k} ({\boldsymbol{x}})}$$

The functions *c*_*k*_(***x***) in (3) measure constraint violations, whereas *β*_*k*_ are the proportionality factors (penalty coefficients) controlling the contribution of particular penalty terms.

Table 1 presents some examples of typical design scenarios which involve antenna reflection response, size, as well as some of field characteristics such as axial ratio or gain. Therein, *f* denotes the frequency, |*S*_11_(***x***,*f*)| represents the modulus of the reflection coefficient at vector ***x*** and frequency *f*, *G*(***x***,*f*) is the antenna gain, *AR*(***x***,*f*) is the axial ratio, and the size is referred to as *A*(***x***) (e.g., footprint area of the substrate the antenna is implemented on). Note that the penalty functions listed in the right-hand-side column represent relative violations of each constraint over the acceptance threshold. The second power is used as it enforces smoothness of *U*_*P*_ as a function of constraint violation at the feasible region boundary, which is numerically advantageous as, at the optimal solution, at least some of the constraints are normally active.

**Table 1:** Exemplary design optimization scenarios for antenna structures.

Design scenario: verbal description	Objective function (1) and constraints	Objective function (3)
Design for best in-band matching within the frequency range *F*	*U*(***x***) = *S*(***x***) = max{*f* ∈ *F* : |*S*_11_(***x***,*f*)|}	*U*_*P*_(***x***) = *U*(***x***)
Design for maximum average in-band gain (in frequency range *F*); ensuring that in-band matching does not exceed − 10 dB in *F*	$U({\boldsymbol{x}}) = \overline{G}({\boldsymbol{x}}) = \frac{1}{F}\int\limits_{F} {G({\boldsymbol{x}},f)df}$ Constraint: $|S_{11} ({\boldsymbol{x}},f)| \le - 10\;{\text{dB}}\;\;{\text{for}}\;\;f \in F$	$U_{P} ({\boldsymbol{x}}) = \overline{G}({\boldsymbol{x}}) + \beta_{1} c_{1} ({\boldsymbol{x}})^{2}$ where $c_{1} ({\boldsymbol{x}}) = \left[ {\frac{{\max (S({\boldsymbol{x}}) + 10,0)}}{10}} \right]^{2}$
Design for minimum in-band axial ratio (in frequency range *F*); ensuring that in-band matching does not exceed − 10 dB in *F*	$U({\boldsymbol{x}}) = {AR} ({\boldsymbol{x}}) = \max \{ f \in F:AR({\boldsymbol{x}},f)\}$ Constraint: $|S_{11} ({\boldsymbol{x}},f)| \le - 10\;{\text{dB}}\;\;{\text{for}}\;\;f \in F$	$U_{P} ({\boldsymbol{x}}) = {AR} ({\boldsymbol{x}}) + \beta_{1} c_{1} ({\boldsymbol{x}})^{2}$ where $c_{1} ({\boldsymbol{x}}) = \left[ {\frac{{\max (S({\boldsymbol{x}}) + 10,0)}}{10}} \right]^{2}$
Design for size reduction of a circularly polarized antenna; ensuring that in-band matching (in frequency range *F*) does not exceed − 10 dB, and axial ratio does not exceed 3 dB	$U({\boldsymbol{x}}) = A({\boldsymbol{x}})$ Constraints: $AR({\boldsymbol{x}},f) \le 3\;{\text{dB}}\;\;{\text{for}}\;\;f \in F$ and $|S_{11} ({\boldsymbol{x}},f)| \le - 10\;{\text{dB}}\;\;{\text{for}}\;\;f \in F$	$U_{P} ({\boldsymbol{x}}) = A({\boldsymbol{x}}) + \beta_{1} c_{1} ({\boldsymbol{x}})^{2} + \beta_{2} c_{2} ({\boldsymbol{x}})^{2}$ where $c_{1} ({\boldsymbol{x}}) = \left[ {\frac{{\max (S({\boldsymbol{x}}) + 10,0)}}{10}} \right]^{2}$ and $c_{2} ({\boldsymbol{x}}) = \left[ {\frac{{\max ({AR} ({\boldsymbol{x}}) - 3,0)}}{3}} \right]^{2}$

### Simulation models of variable-resolution

Variable-resolution models have been used in high-frequency electronics (including antenna engineering) for more than a decade to accelerate simulation-driven design processes^78,87^. Usually, two levels of models are used, often named coarse (also, low-fidelity) and fine (also, high-fidelity). The former may be constructed in the form of an equivalent network representation^79^ or coarse discretization EM analysis^81^. It should be observed, that we use the term “model fidelity” for the level of discretization density of an antenna structure under design in the full-wave simulation model (e.g., finite-difference time-domain, FDTD, or finite element method, FEM).

The pairs of coarse/fine model have been employed in techniques such as space mapping^88^, manifold mapping^89^, or response correction methods (e.g., shape preserving response prediction^90^, adaptive response scaling^81^). Therein, the model of low fidelity is corrected with the use of high-fidelity data accrued in the course of the optimization process and replaces the fine model in the search process. Other uses of the low-fidelity model include initial parameter space pre-screening within machine learning frameworks^69^, as well as variable-fidelity modelling (co-kriging^75^, two-stage Gaussian process regression^91^). The accuracy and evaluation cost of the coarse model fidelity are both important for the efficacy of the variable-fidelity optimization process, yet the appropriate selection of the model is an intricate task^92^.

Low-fidelity models of antenna structures are generally based on coarse-discretization EM analysis as reliable equivalent network or analytical models are hardly available. Reducing the structure discretization in the simulation process (e.g., finite differences time domain, FDTD^93^, etc.) is the major mechanism to speed up the simulation process. Other simplifications include a reduction of the computational domain, neglecting dielectric losses, or considering metal as perfect conductor. In practice, the simplest approach is to control discretization density using a single parameter, e.g., lines per wavelength (LPW) of CST Microwave Studio^94^, which is one of the most widely used commercial EM solvers. In this work, we utilize LPW parameter for setting model fidelity. Observe also, that in our numerical experiments, the number of meshing cells per wavelength and the number of meshing cells per model box edge in CST Microwave Studio are set to the same value (i.e., the current model fidelity *L*).

**Figure 1:**
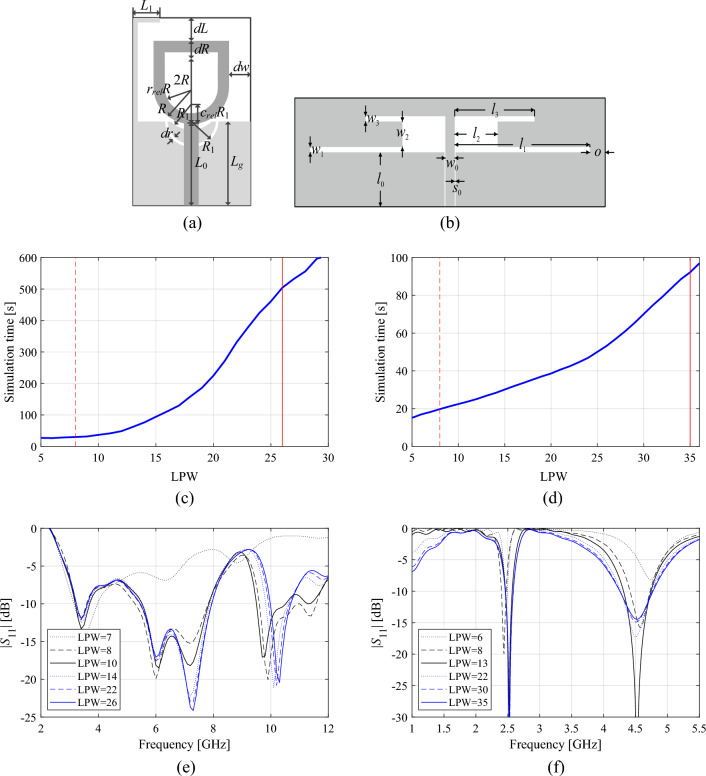
Multi-resolution EM models of a wideband antenna (**a**), and a dual-band antenna (**b**); average simulation time versus LPW for the respective antennas, (**c**), (**d**), respectively; (**e**), (**f**) reflection responses for different discretization densities for the wideband and dual-band antenna, respectively. Vertical lines denote the values of LPW corresponding to the high-fidelity model (—) and the lowest usable low-fidelity model (---).

Consider the antenna structures shown in Fig. 1 along with their reflection responses |*S*_11_|, obtained for different values of the LPW parameter. Larger LPW enlarges the mesh density and, thereby, the accuracy of evaluation, yet, increases simulation time. Both antennas are relatively simple, yet the typical evaluation cost is much higher for the monopole of Fig. 1a because its computational model incorporates the SMA connector^95^. Observe that for some values of LPW, the model usability is questionable as the antenna characteristics it renders is to a large extent misaligned from that of the fine model.

In general, the admissible range of LPW is decided upon visual inspection of antenna characteristics and engineering experience. Here, we will denote by *L*_min_ the lowest value of the control parameter LPW that is acceptable from antenna optimization point of view, which is normally assigned for the model that renders all relevant features of the antenna characteristics (e.g., antenna resonances). The highest value *L*_max_ corresponds to the model of the highest fidelity, which represents the accuracy level satisfactory from the point of view of the designer. The latter can be determined through a grid convergence study, in particular, by finding the value of LPW beyond which no further response changes are observed. 

Having *L*_min_ and *L*_max_, for the sake of acceleration, the optimization process will employ variable-resolution models within the range *L*_min_ ≤ *L* ≤ *L*_max_, where *L* denotes the scalar coefficient controlling the model resolution.

## Nature-inspired antenna optimization with variable-resolution models

This section outlines the incorporation of variable-resolution EM models into population-based nature-inspired antenna optimization. As mentioned in “Introduction” section, perhaps the first attempt to consider multi-fidelity nature-inspired optimization on a generic level was described in the recent paper^83^. Therein, several fidelity adjustment schemes were considered, along with the analysis of the potential benefits of variable-fidelity approach, although the numerical experiments were mainly performed using analytical objective functions. The algorithm discussed in this section is based on a similar idea, whereas variable-resolution EM models of the antenna structures undergoing the optimization process are set up as discussed in “EM-driven design of antennas variable-resolution models” section.

### Generic structure of nature-inspired algorithms

**Figure 2:**
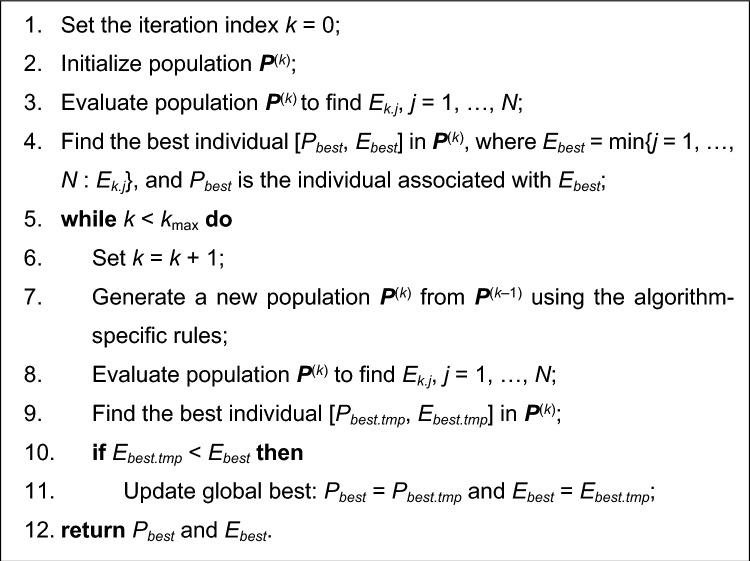
A pseudocode of a generic population-based nature-inspired algorithm processing a population ***P***^(*k*)^ = [*P*_1_^(*k*)^ … *P*_*N*_^(*k*)^] of *N* individuals (particles, agents) throughout a designated number of *k*_max_ generations (Algorithm I).

Consider a generic nature-inspired algorithm presented in Fig. 2. Therein, variable ***P***^(*k*)^ = [*P*_1_^(*k*)^ … *P*_*N*_^(*k*)^] stands for the population (swarm, pack, etc., depending on the type of the algorithm) processed by the algorithm in the iteration *k*. The population size is *N*. The algorithm termination is conditioned by the computational budget, i.e., the prescribed number of iterations *k*_max_. The function *E*(*P*) determines the solution quality; it is to be minimized. *E*_*k.j*_ will be used as a shortcut to *E*(*P*_*j*_^(*k*)^). In the pseudocode of Fig. 2 (Algorithm I), the emphasis is put on the elitism part of the procedure, where the best individual (particle, agent, etc.) is identified and transferred throughout the iterations.

The differences between the various nature-inspired algorithms are pertinent to a construction of a new population ***P***^(*k*+1)^ from the current one. For example, in a genetic/evolutionary type of algorithms^55,56^, a selection of a parent individual from the current population (using partially stochastic operators, e.g., tournament selection^36^) is an intermediate step, followed by the recombination operators, employed to yield the new individuals. Recombination operators are of two major types, exploratory (e.g., crossover^35^), and exploitative (e.g., mutation^35^). In the majority of modern nature-inspired algorithms (PSO^96^, differential evolution^57^, firefly algorithm^42^, and many others), individuals in a population are generally not replaced but rather relocated in the design space using certain rules, typically involving random alterations biased towards the best local and global solutions found so far. For example, in PSO, each particle is associated with its velocity vector, which governs relocation to a new position. The velocity is updated using a linear combination of a random factor, a vector pointing towards the particle’s (personal) best position, and a vector pointing towards the global best. Regardless of the particular set of rules, a vast majority of nature-inspired algorithms can be represented as shown in Fig. 2.

### Incorporating variable-resolution simulation models

Our objective is to accelerate the generic nature-inspired algorithm of Fig. 2 using variable-resolution EM simulations discussed in “Simulation models of variable-resolution” section. Recall that the model resolution is governed using a fidelity factor *L* that can be continuously adjusted between *L*_min_ (the lowest acceptable resolution) and *L*_max_ (high-fidelity model that provides the target accuracy as decided upon by the designer).

As the termination condition of the algorithm of “Generic structure of nature-inspired algorithms” section is based on the maximum number of iterations *k*_max_, the model resolution will be adjusted as a function of the iteration count *k*. We adopt a power-type adjustment scheme (cf.^83^)

4 $$L(k) = L_{\min } + (L_{\max } - L_{\min } )\left[ {\frac{k}{{k_{\max } }}} \right]^{p}$$

where *p* is a control parameter. This scheme offers a sufficient level of flexibility, e.g., for *p* > 1, the model resolution is kept near *L*_min_ for most of the optimization run, and quickly increases towards *L*_max_ when close to convergence. For *p* < 1, only the initial iterations are executed at the low-resolution level, whereas most of the run is carried out close to *L*_max_.

**Figure 3:**
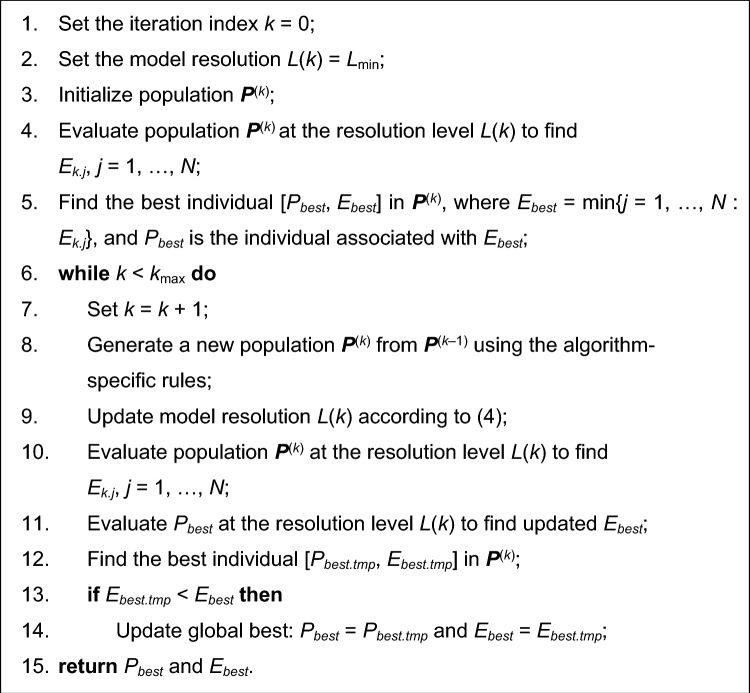
A pseudocode of a generic population-based nature-inspired algorithm incorporating variable-resolution simulation models (Algorithm II). The changes as compared to Fig. 2 include initialization and updating of the current model resolution level (Steps 2 and 9), evaluation of the current population at the current resolution level *L*(*k*) (Steps 4 and 10), as well as re-evaluation of the previously found best individual at the new resolution level before executing Step 13.

Figure 3 shows a pseudocode of a generic population-based nature-inspired algorithm incorporating variable-resolution EM models (Algorithm II). The procedure differs from that of Fig. 2 in several aspects. First, the model resolution equal to *L*_min_ is adopted at the beginning of the algorithm (Step 2). Second, whenever the population members are evaluated (Step 4 and Step 10), it is carried out at the current resolution level *L*(*k*). Subsequently, the model resolution is updated in an automated decision-making procedure according to (4) in Step 9. Finally, the best individual *P*_*best*_ is re-evaluated at the new resolution level before being compared to the best solution extracted from the current population. This is necessary to ensure that the comparison in Step 13 pertains to individuals evaluated at the same resolution level. In other words, the individual that was the best at fidelity level *L*(*k*–1) may not be so at *L*(*k*).

Going back to (4), it is clear that increasing *p* leads to higher computational savings, which can be computed beforehand. Let *T*(*L*) denote the antenna evaluation time at the resolution level *L*. The cost of the algorithm of Fig. 2, executed at the high-fidelity resolution level *L*_max_ can be then simply computed as

5 $$T_{I} = N \cdot k_{\max } \cdot T(L_{\max } )$$

The computational cost of multi-fidelity Algorithm II is

6 $$T_{II} = N \cdot T\left( {L_{\min } } \right) + \left( {N + 1} \right)T\left( {L\left( 1 \right)} \right) + \left( {N + 1} \right)T\left( {L\left( 2 \right)} \right) + \cdots + \left( {N + 1} \right)T\left( {L\left( {k_{\max } } \right)} \right)$$

which gives

7 $$T_{II} \approx (N + 1) \cdot \sum\limits_{k = 0}^{{k_{\max } }} {T(L(k))}$$

This cannot be simplified further as *T*(*L*) is a nonlinear function of *L*, which is not given explicitly. The factor *N* + 1 appears because of the re-evaluation of the best individual (Step 11).

Let us consider the antenna examples shown in Fig. 1. Assuming the population size of *N* = 10, and the maximum iteration number *k*_max_ = 100, which takes into consideration the relations between the fidelity factor *L*, and the average EM simulation time (cf. Fig. 1c, d), one can compute the expected execution times of the algorithm, as juxtaposed in Table 2. It should be observed that both the population size and the maximum number of iterations are low for a typical nature-inspired algorithm, which is to ensure that the computational cost of the optimization process is practically acceptable. As observed in Table 2, the expected costs are still high (about five days for the antenna of Fig. 1a, and one day for the structure of Fig. 1b), even though the considered structures are relatively simple. Yet, in the realm of EM-driven optimization, working out reasonable trade-offs is a practical necessity.

**Table 2:** Computational cost of a generic nature-inspired algorithm for antennas of Fig. 1.

EM model setup		Computational cost of the optimization process (*N* = 10, *k*_max_ = 100)			
		Antenna of Fig. 1a (broadband monopole)		Antenna of Fig. 1b (dual-band dipole)	
		Execution time (h)	Savings w.r.t. high-fidelity-based algorithm (%)	Execution time (h)	Savings w.r.t. high-fidelity-based algorithm (%)
High-fidelity (*L* = *L*_max_)		132.1	–	25.6	–
Variable resolution (cf. (4)) $L(k) = L_{\min } + (L_{\max } - L_{\min } )\left[ {\frac{k}{{k_{\max } }}} \right]^{p}$	*p* = 0.5	82.2	37.7	18.0	29.6
	*p* = 1.0	57.0	56.8	14.5	43.5
	*p* = 2.0	37.7	71.5	11.4	55.4
	*p* = 3.0	29.6	77.6	10.0	60.8

The data in Table 2 also indicates the computational savings that can be achieved with respect to the high-fidelity-based optimization (Algorithm 1), depending on the value of the power factor *p*. Even for *p* = 1, the potential savings may be as high as 50 percent, and increase up to 70 percent for *p* = 3, which is equivalent to a reduction of the execution time by a factor of three or more. While these advantages are attractive, the main questions are whether variable-resolution approach is capable of maintaining reliability, and to what extent computational speedup will be detrimental to the quality of the solutions yielded by the accelerated procedure. These issues will be addressed in “Demonstration case studies” section.

## Demonstration case studies

This section provides the results of numerical validation of the multi-resolution nature-inspired optimization algorithm considered in “Nature-inspired antenna optimization with variable-resolution models” section. The specific instance of the population-based technique, utilized as an optimization engine, is the particle swarm optimizer (PSO)^96^, which is perhaps one of the most popular nature-inspired methods today. The antenna structures employed as verification case studies include a dual-band dipole, a triple-band patch antenna, and two miniaturized broadband monopoles.

The major question to be addressed here is to what extent (if any) the computational speedup obtained by incorporating variable-resolution EM simulations is detrimental to the design quality. This is determined by comparing the results with the single-resolution algorithm employing high-fidelity computational models.

### Test antennas

**Figure 4:**
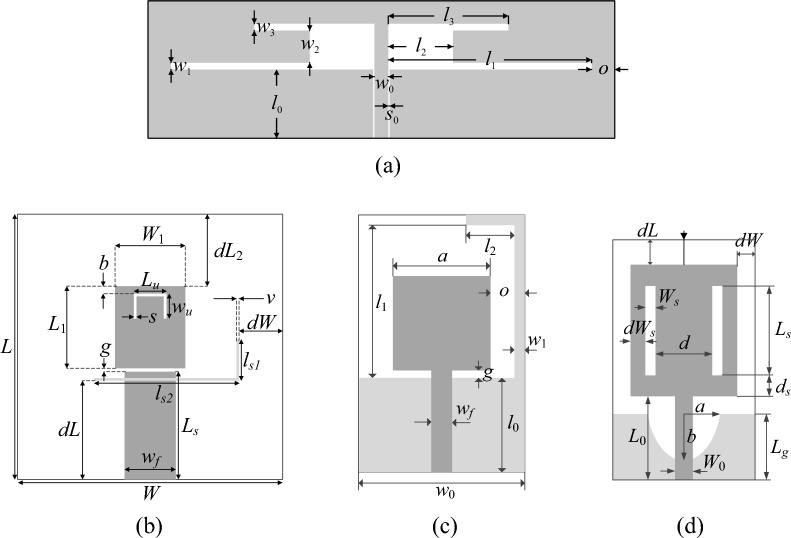
Verification antenna structures: (**a**) Antenna I^98^, (**b**) Antenna II^99^, the light-shade grey denotes a ground-plane slot, (**c**) Antenna III^100^, (**d**) Antenna IV^101^. For Antennas III and IV, the ground-plane metallization is shown using light-shade grey.

The numerical validation is based on four antenna structures that include:

- Antenna I: a dual-band uniplanar dipole antenna^98^ shown in Fig. 4a;
- Antenna II: a triple band U-slotted patch with L-slot defected ground structure (DGS)^99^ shown in Fig. 4b;
- Antenna III: a compact ultra-wideband (UWB) monopole antenna with L-shaped stub^100^ shown in Fig. 4c;
- Antenna IV: a compact ultra-wideband (UWB) monopole antenna with radiator slots^101^ shown in Fig. 4d.

It should be observed that the proposed method is suitable for handling other type of antennas than those presented in Fig. 4. In particular, the formulation of the optimization method is entirely independent of the device under optimization. The only factor that it takes into account is the analytical form of the objective function.

Table 3 provides basic information about the considered structures, including the material parameters of the dielectric substrates the antennas are fabricated on, geometry parameters, target operating frequencies, along with design spaces delimited by the lower and upper bounds for geometry parameters, ***l*** and ***u***, respectively. Observe that the searching spaces are wide, also in terms of the upper-to-lower bound ratios, which are 4.2, 1.5, 19.1, and 4.1, on the average for Antennas I through IV, respectively.

**Table 3:** Verification case studies.

	Case study			
	Antenna I	Antenna II	Antenna III	Antenna IV
Substrate	*ε*_*r*_ = 3.5 h = 0.76 mm	*ε*_*r*_ = 3.2 h = 3.1 mm	*ε*_*r*_ = 3.5 h = 0.762 mm	*ε*_*r*_ = 4.3 h = 1.55 mm
Design parameters	***x*** = [*l*_1_ *l*_2_ *l*_3_ *w*_1_ *w*_2_ *w*_3_]^*T*^	***x*** = [*L*_1_ *L*_*s*_* L*_*u*_* W W*_1_ *dL dW g l*_*s*1_ *l*_*s*2_ *w*_*u*_]^*T*^	***x*** = [*l*_0_ *g a l*_1_ *l*_2_ *w*_1_ *o*]^*T*^	***x*** = [*L*_*g*_* L*_0_ *L*_*s*_* W*_*s*_* d dL d*_*s*_* dW*_*s*_* dW a b*]^*T*^
Other parameters	*l*_0_ = 30, *w*_0_ = 3, *s*_0_ = 0.15, *o* = 5	*b* = 1, *w*_*f*_ = 7.4, *s* = 0.5, *w* = 0.5, *dL*_2_ = *L*_1_, *L* = *L*_*s*_ + *g* + *L*_1_ + *dL*_2_	*w*_0_ = 2*o* + *a w*_*f*_ = 1.7	*W*_0_ = 3.0
Operating bands	8-percent fractional bandwidth w.r.t. center frequencies 3.0 GHz and 5.5 GHz	80 MHz bandwidth centered at operating frequencies 3.5 GHz, 5.8 GHz, and 7.5 GHz	UWB frequency band from 3.1 GHz to 10.6 GHz	UWB frequency band from 3.1 GHz to 10.6 GHz
Parameter space	***l*** = [15.0 3.0 0.35 0.2 1.8 0.5]^*T*^ ***u*** = [50.0 12.0 0.85 1.5 4.3 2.7]^*T*^	***l*** = [10 17 5 45 8 15 9 0.2 4 20 2]^*T*^ ***u*** = [16 25 8 55 12 20 12 0.4 6 24 3]^*T*^	***l*** = [10 10 5 5 2 0.1 0.2]^*T*^ ***u*** = [35 20 15 12 15 10 3]^*T*^	***l*** = [5 5 5 0.2 0.2 5 0.3 0.5 1.0 0.1 0.2]^*T*^ ***u*** = [15 15 15 1.2 8 15 1.5 2.5 5 0.5 0.5]^*T*^

In all cases, the computational models are evaluated using time-domain solver of CST Microwave Studio which utilizes Finite Integration Technique (FIT) as the solver mechanism^111^. The models for Antennas III and IV incorporate the SMA connector^95^. The second row of Table 1 presents the formulation of design problems, i.e., we aim at minimizing the maximum in-band reflection levels.

The simulations have been executed on Intel Xeon 2.1 GHz dual-core CPU with 128 GB RAM. Whereas the code of the proposed optimization algorithm has been written in MATLAB. The particle swarm optimizer and CST simulation software communicate through a Matlab-CST socket, which allows for conveying the design variables found out by PSO to CST and the current value of the discretization parameter *L*, which sets discretization density using in CST Microwave Studio^94^, as well as transferring the simulation results back from CST to PSO.

**Figure 5:**
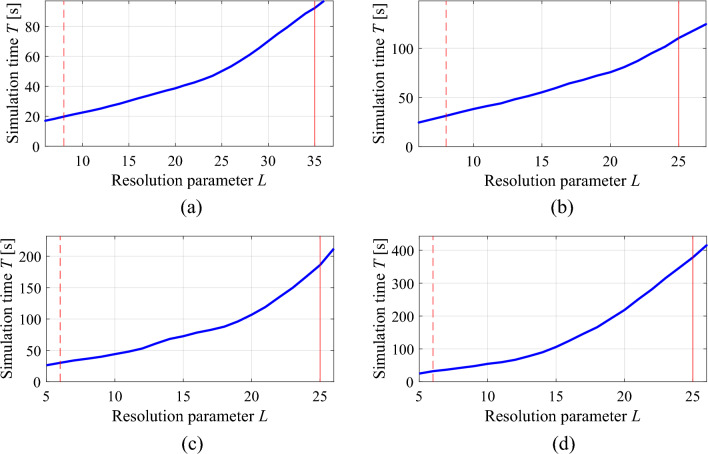
Simulation time vs. EM model fidelity for verification structures of Fig. 4: (**a**) Antenna I, (**b**) Antenna II, (**c**) Antenna III, (**d**) Antenna IV. The minimum usable (---) and the maximum (high-fidelity) (—) values of the resolution parameter *L* are indicated using vertical lines.

Table 4 shows the variable-resolution EM model setup for all considered antennas, including the discretization parameter *L* corresponding to the lowest usable resolution (*L*_min_) and the high-fidelity model (*L*_max_), along with the simulation times. Figure 5 shows the relationships between the model resolution and the average EM simulation time for all considered structures. For the considered structures, the time evaluation ratio between the models of resolutions *L*_max_ and *L*_min_ varies from 3.1 for Antenna I to 11.5 for Antenna IV. Clearly, higher ratio implies higher computational savings that may be obtained through the incorporation of variable-resolution models (cf. Table 2).

**Table 4:** Variable-fidelity EM model setup for verification antennas of Fig. 4.

Antenna	Lowest-fidelity model		High-fidelity model	
	*L*_min_	Simulation time [s]	*L*_max_	Simulation time [s]
I	8	20	35	92
II	8	32	25	114
III	6	28	25	182
IV	6	33	25	378

### Experimental setup and results

Optimization of all verification structures has been performed using the PSO algorithm with the swarm size of *N* = 10, the maximum number of iterations *k*_max_ = 100, and the standard setup of other control parameters, *χ* = 0.73, *c*_1_ = *c*_2_ = 2.05, cf.^96^. The optimization process has been executed using five different scenarios, including the single-resolution version (Algorithm 1) and four variable-resolution versions (Algorithm 2) with the power factor *p* = 1, 2, 3, and 4.

**Figure 6:**
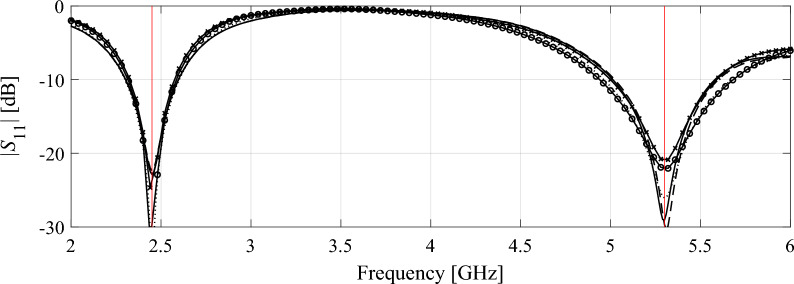
Antenna I: final designs obtained using high-fidelity-based optimization (Algorithm 1) and variable-resolution optimization (Algorithm 2) for representative runs the respective procedures: (—) high-fidelity model, (---) variable-fidelity with *p* = 1, (⋅⋅⋅⋅) variable-fidelity with *p* = 2, (- o -) variable-fidelity with *p* = 3, and (- x -) variable-fidelity with *p* = 4. Target operating frequencies are marked using vertical lines.

**Figure 7:**
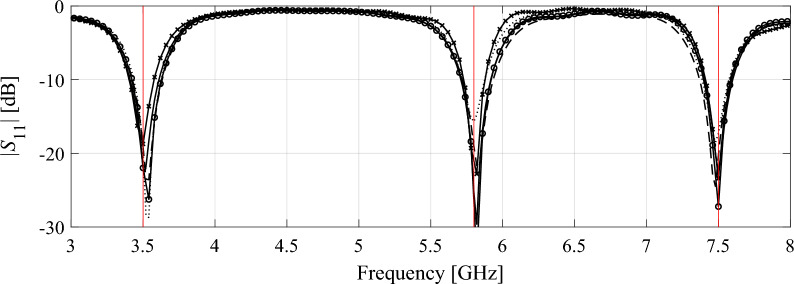
Antenna II: final designs obtained using high-fidelity-based optimization (Algorithm 1) and variable-resolution optimization (Algorithm 2) for representative runs the respective procedures: (—) high-fidelity model, (---) variable-fidelity with *p* = 1, (⋅⋅⋅⋅) variable-fidelity with *p* = 2, (- o -) variable-fidelity with *p* = 3, and (- x -) variable-fidelity with *p* = 4. Target operating frequencies are marked using vertical lines.

**Figure 8:**
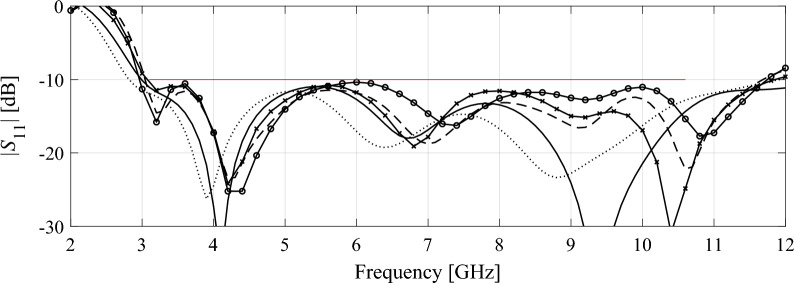
Antenna III: final designs obtained using high-fidelity-based optimization (Algorithm 1) and variable-resolution optimization (Algorithm 2) for representative runs the respective procedures: (—) high-fidelity model, (---) variable-fidelity with *p* = 1, (⋅⋅⋅⋅) variable-fidelity with *p* = 2, (- o -) variable-fidelity with *p* = 3, and (- x -) variable-fidelity with *p* = 4. Target operating bandwidth (3.1 GHz to 10.6 GHz) is marked using horizontal line.

**Figure 9:**
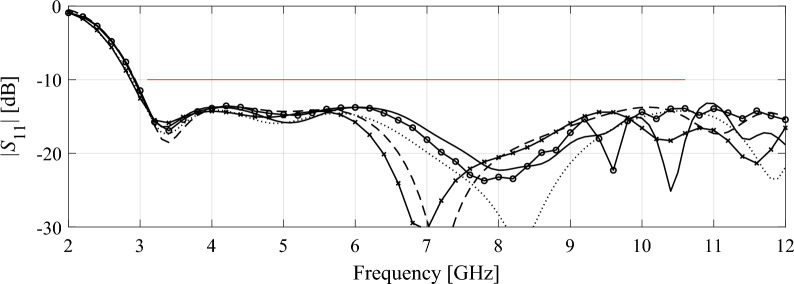
Antenna IV: final designs obtained using high-fidelity-based optimization (Algorithm 1) and variable-resolution optimization (Algorithm 2) for representative runs the respective procedures: (—) high-fidelity model, (---) variable-fidelity with *p* = 1, (⋅⋅⋅⋅) variable-fidelity with *p* = 2, (- o -) variable-fidelity with *p* = 3, and (- x -) variable-fidelity with *p* = 4. Target operating bandwidth (3.1 GHz to 10.6 GHz) is marked using horizontal line.

Tables 5, 6, 7 and 8 gather the numerical results obtained based on fifteen independent runs of each algorithm. In terms of the design quality, we consider the average value of the objective function, which is the maximum in-band reflection level expressed in decibels, as well as its standard deviation as a measure of solution repeatability. Computational efficiency is measured in terms of the overall execution time and the percentage savings with respect to Algorithm 1. Figures 6, 7, 8 and 9 show the antenna responses at the final design yielded in the representative runs of the respective algorithms.

**Table 5:** Optimization results for Antenna I.

Algorithm setup		Execution time [hours]	Savings w.r.t. high-fidelity-based algorithm (%)	Average objective function value [dB]	Standard deviation of objective function [dB]
High-fidelity (*L* = *L*_max_)		25.7	–	− 13.0	2.5
Variable resolution (cf. (4))	*p* = 1.0	14.3	44.4	− 14.7	4.0
	*p* = 2.0	11.2	56.4	− 14.1	3.5
	*p* = 3.0	10.3	59.9	− 15.0	3.2
	*p* = 4.0	9.1	64.5	− 13.6	3.8

**Table 6:** Optimization results for Antenna II.

Algorithm setup		Execution time [hours]	Savings w.r.t. high-fidelity-based algorithm (%)	Average objective function value [dB]	Standard deviation of objective function [dB]
High-fidelity (*L* = *L*_max_)		31.7	–	− 15.7	2.5
Variable resolution (cf. (4))	*p* = 1.0	19.6	38.2	− 18.4	2.1
	*p* = 2.0	16.5	47.9	− 17.9	1.5
	*p* = 3.0	14.5	54.3	− 15.8	2.2
	*p* = 4.0	13.4	57.7	− 14.0	3.0

**Table 7:** Optimization results for Antenna III.

Algorithm setup		Execution time [hours]	Savings w.r.t. high-fidelity-based algorithm (%)	Average objective function value [dB]	Standard deviation of objective function [dB]
High-fidelity (*L* = *L*_max_)		50.9	–	− 10.2	1.2
Variable resolution (cf. (4))	*p* = 1.0	25.4	50.1	− 10.5	1.0
	*p* = 2.0	19.5	61.7	− 10.1	1.2
	*p* = 3.0	17.0	66.6	− 10.3	1.4
	*p* = 4.0	15.4	69.7	− 10.5	1.7

**Table 8:** Optimization results for Antenna IV.

Algorithm setup		Execution time [hours]	Savings w.r.t. high-fidelity-based algorithm (%)	Average objective function value [dB]	Standard deviation of objective function [dB]
High-fidelity (*L* = *L*_max_)		105.1	–	− 13.1	1.6
Variable resolution (cf. (4))	*p* = 1.0	45.6	56.6	− 13.2	1.5
	*p* = 2.0	32.1	69.5	− 13.0	1.6
	*p* = 3.0	26.3	75.0	− 12.9	1.6
	*p* = 4.0	22.5	78.6	− 12.5	1.7

In addition to the data reported in the tables, a Kolmogorov–Smirnov test has been performed for the objective function values obtained during the performed runs of the algorithm to verify the sample normality. In all cases, the null hypothesis that the provided data comes from a normal distribution with the mean and standard deviation as reported in the tables was not rejected at the 5% significance level. The typical *p*-values obtained from the test varies between 0.4 and 0.9. These figures corroborate that the (normalized) distribution of the objective function values between the algorithm runs more or less follows the normal one. On the one hand, this is indicative of the adequacy of the mean and standard deviation as reliable performance indicators. On the other hand, from engineering perspective, this information is of minor significance. What really matters, is that the expected performance of the algorithms under various model resolution adjustment strategies is similar. The detailed analysis will be provided in “Discussion” section.

### Discussion

The results gathered in Tables 5 through 8 allow us to draw several conclusions concerning the efficacy of nature-inspired antenna optimization using multi-fidelity EM models. These can be synopsized as follows:

- The involvement of variable-resolution EM simulations enable significant computational savings. As discussed in “Incorporating variable-resolution simulation models” section, the cost of the optimization process can be controlled using the power factor *p* (cf. (4)). For the antenna cases considered in this work, the reduction of the CPU time ranges from almost 40 percent (for *p* = 1) to over 70 percent (for *p* = 4), with respect to the single-resolution algorithm using the high-fidelity EM model. The average savings across the benchmark set of four antenna structures range from 45 percent (*p* = 1), to 65 percent (*p* = 4).
- Reliability of the optimization process is maintained for variable-resolution algorithm for the power factors of up to *p* = 3; beyond that, one can observe an increase of the standard deviation of the merit function value (indicating degraded repeatability of solutions), or worsening of the average objective function value (Antennas II and IV). An exception is Antenna I, where the average merit function values is better than for single-resolution version even for *p* = 4, whereas repeatability of solution is comparable to all considered values of *p* (while still being slightly worse than for single-resolution algorithm).
- In general, the quality differences between single- and variable-resolution algorithms are relatively small from practical standpoint, which indicates that the incorporation of variable-resolution simulations is indeed advantageous. Assuming—based on the previous observations—the power coefficient *p* = 3, the running time of the optimization process is reduced by a (multiplicative) factor of three as compared to the single-resolution (high-fidelity) approach.
- It should be noted that the PSO algorithm in our experiments has been setup up with a relatively low computational budget of 1,000 objective function evaluations, despite the fact that the considered problems are quite challenging. This is mainly to make the CPU costs of the optimization procedure practically acceptable, which may still be questioned, especially for Antennas III and I. It is expected that increasing the number of algorithm iterations would likely lead to the improvement of solution repeatability.

Overall, it can be concluded that utilization of variable-resolution models enables a significant increase in the computational efficacy of the nature-inspired search without degrading the solution quality, assuming that the model management scheme is selected to allow sufficient time for processing higher-fidelity simulations (here, when using the power factor of up to *p* = 3). Consequently, the presented procedure may be considered a viable alternative to straightforward application of population-based methods in antenna design. Apart from the reduced costs, its advantage is simple implementation, and immunity to both dimensionality and parameter range issues, as opposed, to, e.g., surrogate-assisted frameworks.

## Conclusion

This paper investigated accelerated nature-inspired design optimization of antenna structures using variable-resolution computational models. The analysis of the properties of lower-fidelity EM simulations in terms of the simulation time versus accuracy trade-offs, has been followed by a formulation of a specific optimization framework, involving convergence-driven model management scheme. In particular, the model fidelity has been selected from a continuous spectrum of acceptable resolutions in an automatic manner, with low-fidelity simulations employed at the early stages, and monotonically increasing to the highest assumed fidelity upon algorithm termination.

Numerical validation has been carried out using particle swarm optimizer as a representative population-based routine, and four antenna structures of distinct characteristics (dual-band, triple-band, broadband). The obtained results indicate that sizeable computational speedup of up to almost eighty percent can be obtained without or (for some cases) only slight degradation of the design quality. At the same time, the optimum model resolution management scheme seems to be problem independent. Apart from bolstering the performance, the proposed approach is straightforward to implement and may open new possibilities in terms of making population-based search methods more practical in the context of EM-driven design optimization of antenna systems. Notwithstanding, it should be emphasized that for many real-world antenna systems, individual EM simulation times may be considerably longer than those reported in “Demonstration case studies” section. Thus, despite significant acceleration factors achieved using the proposed approach, the CPU costs of the nature-inspired optimization processes may still be unmanageable. Consequently, the development of even faster methods is a matter of practical necessity. The model management scheme presented in this paper can be viewed as a step towards this direction.

It is also important to emphasize that the presented approach is generic and can be integrated with essentially any population-based optimization engine, including a broad range of nature-inspired algorithms. The results demonstrated based on incorporating the model management scheme into PSO should be viewed as a demonstration example of the concept. The future work will be focused on enabling additional computational savings by extending the model management scheme to differentiate the resolution of the simulation process with respect to other factors, such as the quality of the design.

## Data Availability

The datasets generated during and/or analysed during the current study are available from the corresponding author on reasonable request.
